# The role of personal attitudes of control and responsibility for the uptake of peritoneal dialysis- a national survey among dialysis patients

**DOI:** 10.1186/s12882-021-02303-3

**Published:** 2021-03-24

**Authors:** Isabell Schellartz, Tim Ohnhaeuser, Thomas Mettang, Nadine Scholten

**Affiliations:** 1 University of Cologne, Faculty of Human Sciences and Faculty of Medicine, Institute of Medical Sociology, Health Services Research and Rehabilitation Science (IMVR), Eupener Str. 129, 50933 Cologne, Germany; 2 Kidney Center Wiesbaden, Wiesbaden, Germany

**Keywords:** Hemodialysis (HD), Peritoneal dialysis (PD), Treatment selection, Decision-making, Character, Personality, End-stage renal disease (ESRD), Renal replacement therapy, Desire for control

## Abstract

**Background:**

Although most patients are suitable for both hemodialysis (HD) and peritoneal dialysis (PD), there seem to be differences in the outlook of patients who choose one modality over the other. There is currently limited literature about the impact of patients’ personal attitudes on the decision for PD or HD. In this study, we tried to find out whether there were differences between patients who were on HD and PD in their desire for control and responsibility for their treatment.

**Methods:**

The data were drawn from a nationwide postal survey of 630 HD and PD patients. Patients’ desire for control was measured by scores on the internal locus of control (ILOC) scale. Patients were also asked how important taking responsibility for their dialysis had been for their treatment decision (ITR). Two multivariate logistic regression models, both adjusted for age, were applied to investigate whether there were differences between HD and PD patients in ILOC and ITR. Having one generic measure (ILOC) and one tailored to the dialysis context (ITR) gave the opportunity to investigate if it is a generic personality trait or rather a specific attitude that affects choice of dialysis modality.

**Results:**

PD patients were younger and showed higher ILOC and ITR values. Multivariate logistic regression models adjusted for age confirmed the significant influence of ILOC and ITR on the uptake of PD. The odds ratios for being in the PD group were 1.53 for ILOC (*p* = 0.030; 95% CI 1.04–2.25), 1.49 for ITR (*p* = 0.019; 95% CI 1.07–2.07), and 0.95 (*p* = 0.000; 95% CI 0.94–0.97) for age in both models.

**Conclusions:**

Our analysis shows the impact of personal attitudes on the uptake of PD. Participants who generally want to keep control of their lives and take responsibility for their dialysis treatment tended to choose PD. As PD is a home dialysis treatment that requires patients to participate and contribute, it is beneficial if patients’ personalities support the treatment procedure. Having two completely different treatment options that suit to different personalities gives us the opportunity to consider the relationship between personal attitudes and choice of dialysis modality.

**Trial registration:**

The MAU-PD study is registered at the German Clinical Trials Register.

DRKS-ID: DRKS00012555.

Date of Registration in DRKS: 2018/01/04.

## Background

Hemodialysis (HD) and peritoneal dialysis (PD) are considered equivalent options for the treatment of end-stage renal disease (ESRD) in terms of survival rates [1, 2]. Although it has been reported that patients with PD show better outcomes regarding their quality of life [3–5], Germany shows a low PD-ratio of 6% [6]. The compulsory statutory health insurance (SHI) in Germany covers about 90% of the population [7]. This SHI reimburses both, in-center and home-based dialysis treatment options as well as the transport to the ambulatory dialysis center [8]. There is also a legal obligation to inform patients about different treatment options [9]. Looking at patient-related characteristics, the international literature shows, that due to only a few absolute contraindications against PD, most ESRD patients are eligible for both modalities [10, 11]. Nevertheless, several studies have identified differences in educational level and gender between HD and PD patients [5, 12–15], and the majority of prior research indicates PD patients to be younger [2, 3, 12–17]. Other patient-related factors, like personality traits or attitudes, and their role in the context of dialysis modality choice have hardly been investigated.

One aspect of personality is the desire for control. If a person expects an event to be the result of his or her own behavior, this is called a belief in internal control. Hence, persons with a strong belief in internal control are convinced they determine events by their own knowledge and skills [18]. Among others, this desire for control has been used in medical-sociological research contexts to explain medical decisions or medical events [18–22].

There is limited, mainly qualitative evidence about the role of the desire for control in the context of dialysis patients. Studies have shown the relevance of the desire for control when making a decision on renal replacement therapies [23–25]. PD patients often suggest that control and autonomy were causal for their treatment choice [26, 27] whereas HD patients or former PD patients after the transition to HD reported a loss of control [28, 29]. In addition, pre-dialysis patients emphasized their need to be personally responsible for their treatment decision-making process [30].

The aim of the present study is to investigate the impact of patients’ desire for control and their view on the importance of taking responsibility for their own dialysis treatment on the decision for PD. Deepening existing qualitative examinations, the results contribute further insights through a representative quantitative survey using a validated instrument [19].

## Methods

### Survey

As the majority of the German population is contracted with SHIs [7], we collaborated with two large SHIs covering 6.6 million insurants [31, 32]. DAK-Gesundheit (Deutsche Angestellten-Krankenkasse) and SBK (Siemens Betriebskrankenkasse) selected adults (≥18 years of age) who were dialyzing among their insurants. These insurants were contacted for a nationwide postal survey. The questionnaire, study information, and a franked envelope were prepared by the study group. Then the SHIs mailed them to their insurants at the end of 2018/beginning of 2019. The study group did not know the addresses of the insurants. Participants returned the completed questionnaire to the study group. Due to this anonymous survey design, the participants gave their informed consent to participate and publish the summarized results by completing and returning the questionnaire. Participants were informed about this procedure in the written study information. The procedure follows good clinical practice and the Declaration of Helsinki.

Through this procedure, 2095 patients were approached. After a one-time reminder, regardless of whether the survey had been completed by that time or not, 964 participants responded (46% response rate). The survey was carried out as part of the MAU-PD study (German Clinical Trials Register ID: DRKS00012555), which aims to assess the reasons for the low PD ratio in Germany from different perspectives with a mixed-methods design [33].

### Measures

In order to address the desire for control in the questionnaire, we used the validated 3-item short scale “Internal locus of control” (ILOC), which is based on the concept of belief in internal control [19]. The scale’s three statements refer to the patient’s level of taking responsibility, making decisions on their own, and ability to assert themselves, which were added up to a mean ILOC value. Answer categories were on a 5-point Likert scale from 1 “strongly disagree” to 5 “strongly agree”. Due to their positive verbalization, high ILOC values indicate a strong belief in internal control [19]. We only calculated the ILOC scale means for participants who answered all 3 questions.

Besides this general attitude of a belief in internal control, respondents were explicitly asked how important it was for them to take responsibility for their decision regarding dialysis modality (ITR). This question was carried out from a number of items asking, “how important were the following aspects for your decision regarding dialysis modality?” For all of those items, we used an answer scale from 1 “not important at all” to 4 “very important”. Because in this set of questions, aspects were mentioned that did not affect every participant (e.g., the opinion of relatives), an additional answer category of “not applicable” (N/A) was added. Hence, there was the 4-point Likert scale with the additional answer category N/A for ITR. These items were developed by the study group. Furthermore, participants were asked about their current dialysis modality, if the modality had changed in the past, and about sociodemographics like age, sex, and school education.

Having one generic measure (ILOC) and one tailored to the dialysis context (ITR) gives the opportunity to investigate both: Is there a generic personality trait or is it rather a specific attitude that affects the decision for PD choice of dialysis modality?

### Analysis

This analysis aims to investigate the difference between in-center HD patients and PD patients (CAPD or CCPD). Focusing on the role of personal attitudes in decision-making for PD, we also categorized participants as PD patients who had initially decided for PD and then later changed from PD to HD. In order to compare effects of the general ILOC and the dialysis-specific ITR within the same study population, participants with missing values in one of these variables or those without information about their dialysis modality were excluded. Participants who stated that ITR was N/A were also excluded, because N/A is an alternative category to the remaining Likert scale, and it is not a direct answer to the question. For a reliability analysis, Cronbach’s alpha was calculated for ILOC.

Univariate differences between HD and PD patients were analyzed regarding age, sex, and school education as well as ILOC and ITR. The comparison of the ILOC values of HD and PD patients indicates whether there is a difference between those two groups regarding this generic personality trait; in contrast, ITR differences reflect whether there is a specific attitude, that is relevant for the decision for PD. Depending on the variables’ nature and their distribution, Wilcoxon-Mann Whitney and Chi-square tests were used. Taking a possible confounder into account, age and ILOC were combined in a multifactorial logistic regression model for the dialysis modality group (HD or PD; Model A). In addition, the association of ITR and the dialysis modality group was examined and also adjusted for age (Model B). In order to investigate whether participants with a high desire for control also have a higher desire to take responsibility for their own dialysis treatment, a Spearman’s rho correlation between ILOC and ITR was calculated. We also investigated ILOC differences between ITR responders and participants who ticked N/A with ITR to assess the external validity of our results.

Stata 16.1 was used for statistical computations.

## Results

### Study population

**Fig. 1:**
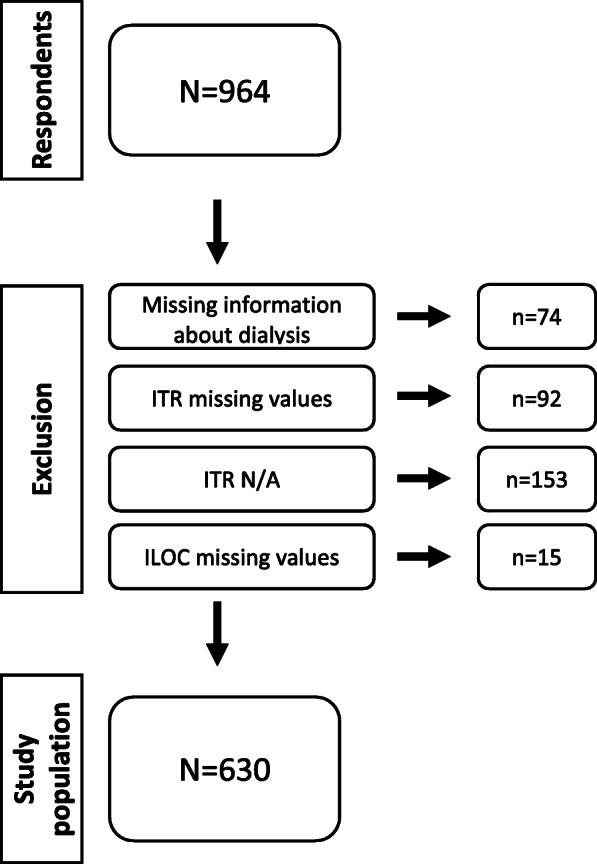
Excluded participants

As shown in Fig. 1, 92 of the excluded patients did not answer ITR, and 153 chose N/A, of which 85 and 149 were HD patients, respectively.

A total of 630 dialysis patients were included in this analysis. Initially, 90% (*n* = 565) started with in-center HD and 10% (*n* = 65) started with PD (CAPD, CCPD). Of those patients who started with PD, 37 (57%) later changed to in-center HD and one patient to home-based HD (2%). At the time of the survey, the proportion of patients currently dialyzing via PD was 4.3%. Participants were on average 68 years old (range of 22–96), and 40% were female. Table 1 provides further information about the study population. PD patients were significantly younger than HD patients. There were no differences regarding sex or level of school education between the HD and PD patients.

**Table 1:** Participant characteristics

	HD (***n*** = 565)	PD (*n* = 65)	Prob > |z|
**Age, mean (min-max)**	69 (22–96)	60 (30–86)	0.000
**Male, n (%)**	343 (61)	36 (55)	0.406
**School education, n (%)**			
No school qualifications	13 (2)	2 (3)	0.398
Basic school qualification	244 (43)	23 (36)	
Extended secondary school diploma	193 (34)	25 (39)	
A-levels	111 (20)	14 (22)	
**ILOC, mean ± std. dev.** Answer scale: 1–5	4.0 ± 0.76	4.3 ± 0.68	0.020
**ITR, mean ± std. dev.** Answer scale: 1–4	3.2 ± 0.9	3.5 ± 0.8	0.010

*HD* Hemodialysis, *PD* Peritoneal dialysis, *ILOC* Internal locus of control, *ITR* Importance of taking responsibility, *std. dev* standard deviation

### ILOC

Reliability analysis of ILOC revealed a Cronbach’s alpha of 0.79. As displayed in Table 1, PD patients show a significantly higher ILOC than HD patients (4.3 vs. 4.0, *p* = 0.020). As age was found to be the only sociodemographic confounder in our study, a multivariate logistic regression for modality choice (HD vs. PD) with ILOC and age as independent variables was built (Model A; see Table 2). In this model, both age and ILOC showed significant effects on the uptake of PD. The likelihood of being in the PD group was 5% lower with every additional year of life (*p* = 0.000; 95% CI 0.94–0.97). With a one-point higher ILOC on the 5-point Likert scale, the likelihood of being in the PD group was 53% higher (*p* = 0.030; 95% CI 1.04–2.25). Pseudo R^2^ of Model A is 0.074.

**Table 2:** Results of the multivariate logistic regression models on the uptake of PD (Models A and B)

	Model A			Model B		
	Odds Ratio	Prob>|z|	CI	Odds Ratio	Prob>|z|	CI
**Age**	**0.95**	**0.000**	**0.94–0.97**	**0.95**	**0.000**	**0.94–0.97**
**ILOC**	**1.53**	**0.030**	**1.04–2.25**	–	–	–
**ITR**	–	–	–	**1.49**	**0.019**	**1.07–2.07**
**Cons**	0.39	0.363	0.05–2.94	0.68	0.636	0.14–3.38

*CI* Confidence Interval, *ILOC* Internal locus of control, *ITR* Importance of taking responsibility, *Cons* constant, *Prob* Probability, *PD* Peritoneal dialysis

### ITR

Taking responsibility for their treatment was rated as very important by 50% of the participants. For 6%, this was not important at all. Table 1 shows the mean ITR values of HD and PD patients; Table 3 shows the distribution. On average, PD patients rated ITR higher than HD patients (3.5 vs. 3.2, *p* = 0.010). In particular, the percentage of participants who stated that taking responsibility was very important for them was higher in the PD group than in the HD group (66% vs. 48%). As age was found to be the only sociodemographic confounder in our study, another multivariate logistic regression for modality choice (HD vs. PD) with ITR and age as independent variables was built (Model B). Both age and ITR are significant in this model (see Table 2). The likelihood of being in the PD group decreased by 5% with every additional year of life (*p* = 0.000; 95% CI 0.94–0.97). Patients who rated ITR one point higher on the 4-point answer scale had a 49% higher likelihood of being in the PD group (*p* = 0.019; 95% CI 1.07–2.07). Pseudo R^2^ of Model B is 0.077.

**Table 3:** Association of ITR with ILOC and dialysis modality

	HD		PD	
ITR	n [%]	Mean ILOC-values	n [%]	Mean ILOC-values
**Very important**	272 [48.3]	4.3	43 [66.15]	4.4
**Rather important**	178 [31.5]	3.9	13 [20.0]	3.9
**Not very important**	76 [13.5]	3.6	7 [10.8]	4.0
**Not important at all**	38 [6.7]	3.7	2 [3.1]	4.2

*HD* Hemodialysis, *ITR* Importance of taking responsibility, *ILOC* Internal locus of control, *PD* Peritoneal dialysis

### ILOC and ITR

Table 3 shows mean ILOC values depending on ITR answer categories. Spearman’s rho of ILOC and ITR is 0.31 (*p* = 0.000), meaning 10% of the variability of both parameters is shared. As this study aims to investigate and quantify the impact of ILOC and ITR on the uptake of PD and there were limited opportunities for the adjustment of confounders, a combined model was not necessary to answer the research question.

Due to the high proportion of excluded participants who answered N/A in the ITR, a Wilcoxon-Mann Whitney test was conducted to investigate ILOC differences between N/A responders and our study population. Our study population stated higher ILOC values than N/A responders (4.1 vs. 3.8; *p* = 0.007). Significantly more HD patients ticked N/A in the ITR (*p* = 0.003).

## Discussion

Our study shows that the desire for control, operationalized by the ILOC scale, has a significant impact on the uptake of PD. Furthermore, the extent to which it is important for the participant to take responsibility for their own dialysis treatment (ITR) affects the decision for HD or PD. First, univariate tests show that ILOC and ITR vary significantly over HD and PD patients. Second, multivariate models with adjustment for age (Models A and B) confirm this effect. In both models, the confounder of age keeps its impact.

Both multivariate models (A and B) revealed that the effect of ILOC or ITR cannot be solely explained by the younger age of the PD patients. The odds ratio of 1.53 in Model A means that people one level higher on the 5-point ILOC Likert scale have a 53% higher chance of being in the PD group (adjusted for age). A one-level-higher ITR on the 4-point answer scale means a 49% higher chance of being in the PD group. The effect of age is similar in both models. Comparing the odds ratios of ILOC and ITR, makes clear that they have a similar impact.

A total of 153 patients ticked N/A for ITR. They were excluded because N/A is an alternative category to the remaining Likert scale, which means that the respondent has not provided a direct answer to the particular question. Thus, the N/A response category cannot be included in an analysis using a Likert scale. However, in this context N/A could mean that taking responsibility was not an issue and therefore not important at all. The significant lower ILOC values in N/A responders in the ITR and the positive correlation between ITR and ILOC underline this assumption. Since responders who answered N/A were mainly HD patients, we assume that the effect of ITR could have been even larger if they had been included in the analysis. This has to be interpreted with caution. Hence, the chosen methodological approach offers a conservative estimation of the effect of ITR.

Although some of the previous qualitative studies did not differentiate between the dialysis modalities, they established the relevance of the desire for control when deciding between different renal replacement therapies [23, 24]. PD patients often stressed aspects of autonomy and control that were causal for their decision [26, 27], whereas HD patients reported a loss of control because of the feeling they could not manage their life any longer [28]. This became particularly clear in quotes from semi-structured interviews [24, 26, 28, 29]. Our study, using the validated ILOC scale [19] and a larger sample (*n* = 630) is the first quantitative examination that confirms this association.

The uptake of PD is generally dependent on the socioeconomic context of the healthcare system, reimbursement policies, the PD provision by nephrologists or staff perceptions regarding a candidate’s suitability for PD [34–37]. Despite the SHI reimburses all dialysis options, Germany historically shows a low PD utilization. The dialysis providers also support the uptake of PD. Hence, the framework conditions for a higher PD utilization give the impression to be in place and there seem to be other factors on the providers’ level. Researchers have been trying to grasp for years, why these factors are strong drivers especially in Germany. Nevertheless, as PD is a home dialysis treatment that requires patients to participate and contribute, it is beneficial if patients’ personalities make them suited to the treatment. Besides patients’ personal conditions like poor mobility, comorbidities [38], a lack of space for PD supplies at home [39] or the involvement of partners [40], nephrologists should be aware of taking patients’ personality into account to get an idea whether PD could be an option for them. A review of the barriers in dialysis modality choice showed that pre-dialysis education programs and decision aids can help to enable patients to make an autonomous and confident decision when selecting a dialysis modality [41]. The authors explain this by patients’ lack of knowledge, which reduces the level of confidence and control [41]. Structured patient education programs are also associated with increased utilization of PD [4, 42, 43]. Of course, generic personality traits cannot be fundamentally changed by information or patient education, but the usage of pre-dialysis education programs could help to reduce fear and encourage patients to be autonomous and confident enough to opt for PD. At an early stage of the dialysis modality decision, this approach may enable an informed decision considering patients’ personal attitudes.

### Strengths and limitations

The German SHI is compulsory and its insurance companies are obliged to contract with every person and, thus, has a broad collective of insurants all over Germany [44]. Due to the cooperation with DAK-Gesundheit and SBK covering 6.6 million insurants [31, 32] and the high response rate of 46% this study has a representative sample with minimized risk of a selection bias.

Our study population consisted of 630 respondents out of about 75,000 German dialysis patients receiving ambulatory dialysis treatment reimbursed by the SHI [6]. The mean age in the study population (HD = 69, PD = 60) was marginally higher than the general dialysis patient population in Germany (HD = 68, PD = 58) [6]. The proportion of women included in this analysis was similar to the national average (39.8% vs. 39.3%) [6]. We show a lower percentage of prevalent patients dialyzing via PD in our sample (4.3% vs. 6.1%) [6]. The difference may be related to the fact that the basis for calculation varied. The underlying report on quality in dialysis is based on the number of conducted dialyses billed (annual prevalence). The SHIs selected their insurants on dialysis for the survey (point prevalence). Annual and point prevalence are thus difficult to compare. However, because we focus on the role of personality in decision-making for PD, we assessed it as adequate to investigate the differences between HD patients and those who had primarily decided for PD, no matter if they later changed from PD to HD. The exclusion of participants choosing N/A in ITR excluded relatively more HD than PD patients. This may have led to a selection bias.

A Wilcoxon-Mann Whitney test showed N/A responders in the ITR show slight but significant lower ILOC values compared to our study population. This could indicate a limited external validity of our results, because we excluded a certain group from our study population with a significantly different attitude. Since the N/A responders in the ITR are also more likely to be HD patients, this may also confirm our assumption that the N/A category even could be treated as “not important at all”. As we cannot clarify this conclusively, we assume that the N/A category has to be treated as special types of missing values that we cannot simply address with imputation techniques. Therefore, the exclusion of N/A responders were methodologically necessary to compare the effects from models A and B. Thus, we chose a conservative approach to estimating the effects and accepted the risk of a limited external validity.

In surveys, we often see a social desirability response set. Especially with the ILOC scale, the authors state this form of response bias, because personalities with a high desire to take responsibility or be assertive are desirable in a competitive society [19]. Nevertheless, there are no hints in our sample that this bias varies between HD and PD patients. If there is a general response set in the direction of high ILOC values, this is probably similar in the HD and PD groups.

The multivariate models were adjusted for age. Adjusting only for one confounder means a limited risk adjustment. There are other, patient-related and non-patient-related factors expected to affect the uptake of PD. This is also reflected in the Pseudo R^2^, which shows limited predictable variance of 7 to 8%, respectively. Due to the anonymous survey design, patients self-reported their information, and thus we had limited opportunities to collect all possible confounders. We examined the popular confounders of age, sex and school education. Looking at these factors, patients in our study population only varied in age. Hence, we decided to adjust only for age in our model.

## Conclusions

Our analysis showed that aspects of the patients’ personality play a role in the uptake of PD and should receive more attention. Participants who generally want to keep control of their lives and take responsibility for their dialysis treatment tended to choose PD. As PD is a home dialysis treatment that requires patients to participate and contribute, it is beneficial if patients’ personalities make them suited to the treatment procedure. Having two completely different treatment options, which are appealing to different personalities, gives us the opportunity to consider personal attitudes in decision-making for dialysis modality.

## Data Availability

The data support the findings of this study are available on request, with permission of the study group, DAK Gesundheit and SBK.

## Abbreviations

CI: Confidence interval
DAK: Deutsche Angestellten-Krankenkasse
ESRD: End-stage renal disease
HD: Hemodialysis
ILOC: Internal locus of control
ITR: Importance of taking responsibility
N/A: Not applicable
PD: Peritoneal dialysis
Prob: Probability
SBK: Siemens Betriebskrankenkasse
SHI: Statutory health insurance
Std. dev.: Standard deviation
